# Suicide and Its Legal Implications in Pakistan: A Literature Review

**DOI:** 10.7759/cureus.1665

**Published:** 2017-09-08

**Authors:** Sadiq Naveed, Tooba Qadir, Tayyaba Afzaal, Ahmed Waqas

**Affiliations:** 1 Psychiatry, KVC Prairie Ridge Hospital; 2 Dow University of Health Sciences, Dow University of Health Sciences, Karachi, Pakistan; 3 Psychology, Government College University, Lahore, Pakistan; 4 Department of Psychiatry, CMH Lahore Medical College and Institute of Dentistry

**Keywords:** suicide, risk factors, islam, pakistan

## Abstract

In recent decades, great strides have been made in understanding the science of suicide. Thus, it is imperative that Pakistani legal systems bridge the gap between Pakistani law and science. For instance, recent discoveries in public health, psychology, and neurobiology have shaped the etiological model of suicidal behavior and highlighted the high preponderance of certain psychiatric patients towards suicide. We present here a brief overview of psychiatric evidence implicated in suicides to better inform the Pakistani legal system of advances in the psychiatric literature.

## Introduction and background

Situated in South Asia, Pakistan is geographically small but is the sixth most populous country in the world. The majority of Pakistan’s population is Muslim (97%), and it is also home to some important religious minorities, including Christians, Hindus, Sikhs, Buddhists, and Zoroastrians [1]. In Islam, suicide is considered a sin [2-4], and because Pakistani law is based on Islamic values, suicidal behavior and attempted suicide are considered criminal offenses punishable by law and liable to fines and/or imprisonment [1].

Due to the sensitive nature and social and legal taboos associated with suicidal behaviors, such behaviors are often underreported in Pakistan. Despite being a harsh reality, suicide and suicidal behaviors have not been well studied, and very little research has been conducted in this field.

## Review

To help elucidate the scientific and medical implications of suicide and enhance the current understanding of suicide within the Pakistani legal system, this article discusses several important aspects of suicide, including recent studies on the neurobiology of suicide, the interplay of religion and suicide, and current Pakistani suicide data. The authors also discuss the legal implications of suicide in Pakistan and evaluate the current trends in suicide and mental health in Pakistan.

Neurobiology of suicide

Suicide is a complex phenomenon resulting from several risk factors, including a family history of suicide, genetic loading, psychosocial stressors, underlying psychiatric illnesses, traumatic life events, personality traits, and cognitive distortions [5]. Given the numerous risk factors, suicide research has been increasingly focused on biological markers for suicide because the early recognition of suicidal behaviors can be valuable for the prevention and treatment of suicide [6].

Several studies associate suicide attempts with a dysregulation of the hypothalamic-pituitary-adrenal (HPA) axis, making HPA a top candidate as a biomarker. Additional findings are correlated with neuroinflammatory indices, glutamatergic function, and neuronal plasticity at the cellular and circuitry level, as well as impairments in stress response systems [7].

A dysregulated HPA axis is also linked with other systems involved in suicide, such as the serotonin, opioid, and glutamate systems, inflammatory pathways, lipid status, neuroplasticity, and neurogenesis [7].

Studies evaluating inflammatory markers in suicidal patients revealed persistently elevated levels of certain neurochemicals in those patients [6]. The inflammatory cytokine interleukin (IL-6) was most frequently associated with suicidality and found in the cerebrospinal fluid (CSF), blood, and postmortem brain [6]. One study reported higher levels of Interleukin-6 (IL-6) in the CSF in violent suicide attempters compared with nonviolent attempters and in cases that resulted in future suicide completion [6]. Low plasma Interleukin-2 (IL-2) was observed in some studies of suicide attempters, while tumor necrosis factor-α, interferon-γ, transforming growth factor-β, Interleukin-4 (IL-4), and soluble IL-2 receptors had variable results [6].

A study comparing vitamin D levels in suicide attempters, nonsuicidal patients with depression, and healthy controls showed suicide attempters to have significantly lower mean levels of vitamin D than depressed non-suicidal patients and healthy controls. Furthermore, vitamin D and pro-inflammatory cytokines in the psychiatric patients were inversely proportional to low vitamin D levels and were associated with higher levels of IL-6 and Interleukin-1 (IL-1) in the blood [8]. While research shows an assortment of connections between varied biomarkers, larger and more definitive studies are required to understand the interaction between different contributing elements and to study potentially modifiable risk factors [6].

Religion and suicide

Widespread implementation of religion and adherence to religious practices have been established as a protective factor against suicide [3]. Most well-known religions in the world, including Christianity, Islam, Hinduism, Buddhism, and Judaism, condemn the practice of suicide [1]. In fact, the World Health Organization (WHO) reported in 2002 that countries with the highest suicide rates were those where religious activities were banned or discouraged (e.g., the former Soviet Union and communist countries of Eastern Europe) [9]. Suicide rates were lower in countries practicing Hinduism, Buddhism, and Christianity, with the lowest rates reported in Muslim-majority countries [3].

Islam is the second largest religion in the world, accounting for more than 20% of the world’s population, practiced in over 56 Muslim-majority countries in Asia and Africa [3]. Like many other religions, Islam denounces suicide but also goes a step further to declare it a strictly forbidden “sin,” which Islamic scholars deem unforgivable [2]. Some verses in the Quran specifically condemn violent acts, including suicide and state, “you should not kill yourself because God has been merciful to you” (4:29). Islam asserts that birth and death are divine decrees, and therefore, even thinking about suicide is prohibited as it indicates one’s lack of commitment and degree of despair [10-11]. This specific commandment plays a great role in the prevention of suicides in members of Muslim communities [12]. The Quran refers to suicide as “self-murder” (i.e., Qatl-e-Nafs) and “cutting of the throat” (i.e., intihār) without actual thematic references to it. Not only do the prophetic teachings forbid suicide and condemn the perpetrator to eternal retribution in the form of never-ending repetitions of the act and the anguish of the mode of suicide, but they also explicitly discourage the mental act of wishing for death [13]. The suicide rates in Muslim-majority countries, accordingly, have been reported in several studies to be substantially lower compared to that of non-Muslim-majority countries [2, 4]. One study found the rates of suicide in seven Muslim-majority countries, including Iran, Pakistan, Kuwait, Iraq, Syria, Jordan, and Egypt, were significantly lower when compared to Western countries [14]. Even in non-Muslim countries, the rates of suicide in Muslims inhabitants have been lower than non-Muslim inhabitants [1]. There is a reason to believe, however, that these statistics may not correctly represent true suicide rates in Muslim populations [1]. One study reported that the rates of attempted suicide were not lower in Muslims compared to non-Muslims [4]. Nevertheless, the lower reported suicide rates may have led researchers to the incorrect assumption that studying suicide and parasuicide in the Islamic world is futile [1]. This emphasizes the need for further research on suicide and attempted suicide in Muslim communities and Muslim-majority countries.

Suicide in the context of Pakistan

According to 2008 WHO statistics, worldwide deaths by suicide amounted to 782,000, representing 1.4% of the total mortality and 15% of reported injury mortality. The worldwide suicide rate is estimated at 11.6 per 100,000 inhabitants. However, the WHO has only been able to collect records from approximately half the countries in the world [15]. Pakistan reported no suicide mortality data to the WHO [1]. No official numbers are available, and national suicide rates are neither known nor included in the annual mortality statistics [4,16].

Research on suicide and attempted suicide in Pakistan is sporadic; only three studies were conducted on suicide, and six studies were conducted on attempted suicide [17-20]. Ahmed and Zuberi estimated suicide rates in the city of Karachi from 1959 to 1963 as 0.72 per 100,000 and from 1974 to 1978 as 0.11 per 100,000 [18].

While official national rates are unavailable, suicide rates in the six largest cities in Pakistan have been calculated. Crude rates differ from a low of 0.43 per 100,000 per year (the average for 1991 to 2000) in Peshawar to the highest rates of 2.86 per 100,000 per year for Rawalpindi (in 2006). Other cities fall somewhere between the two extremes on the spectrum: Karachi, 2.1 per 100,000 (1995 to 2001); Lahore, 1.08 per 100,000 (1993 to 95); Faisalabad, 1.12 per 100,000 (1998 to 2001); and Larkana, 2.6 per 100,000 (2003 to 2004) [21].

Gender-specific suicide rates show that, for men, the highest rate is 5.2 per 100,000 in Rawalpindi, while the highest rate for women is 1.7 per 100,000 in Larkana. The highest age and gender-specific rates for men and women are in the 20 to 40-year age group: 7.03 per 100,000 for men and 3.81 per 100,000 for women in Larkana. More than 10 years ago, a non-governmental organization reported 5,800 suicides in nine months (from January to September of 2006) [16]. Despite these very low rates, suicides occur in all non-reporting countries, including Pakistan, and evidence suggests that the actual suicide rates are at least equal to, if not greater, than the official rates [20]. There is also evidence that suicide rates are steadily increasing [22]. The discrepancy between actual rates and reported rates probably exists because there is not a well-organized surveillance system, and the figures are collected from police data, which usually significantly underestimate the actual rates in the official police records [20, 23].

The studies of suicide and attempted suicide are further complicated by a multitude of existing cultural, social, legal, and religious aspects, leading to an underestimation for both suicides and suicide attempts. Therefore, the true magnitude of the problem is difficult to determine. Completed and attempted suicidal behavior may be concealed either by a lack of reporting or misclassifying the event, and thus, the rates of completed and attempted suicide in Islamic countries like Pakistan might be misleading [4].

Legal implications of suicide in Pakistan

Given Pakistan’s Muslim-majority population, Pakistani laws are built on Islamic principles and guidelines. By the rules of Islam, suicides and attempted suicides are considered criminal offenses under the Pakistan Penal Code 309 of the Criminal Procurement Act, punishable by imprisonment and/or subject to financial penalty up to Rs 10,000 [20]. By law, suicide and attempted suicide must be reported to the police for further investigation. Such cases must be evaluated at government-designated medico-legal centers (MLCs) specially authorized to receive forensic cases [1,16].

Prosecution due to criminal suicide offenses is rare, and confidentiality around such a sensitive issue leads to the concealment of facts. People avoid going to MLCs due to the fear of harassment, embarrassment, and stigmatization by the police and their society. Due to the complications associated with police reporting, cases are taken to private hospitals where they are neither explored or brought to the authorities. Consequently, the phenomenon of suicide and parasuicide remains undiagnosed and understudied [1, 20]. In rare cases where coexisting psychiatric disorders are the cause of the suicide attempt, patients may be provided access to psychiatric treatment rather than penalized [21].

Trends of suicide and mental health associations in Pakistan 

Research conducted over the last 80 years has shown that suicidal behaviors occur in almost all cultures and societies at various stages of life, and suicidal behavior is predisposed by a variety of circumstances [20]. In Pakistan, a complex interface of socio-cultural and legal perspectives surround the act of suicide. Suicide is perceived as a disgraceful act to be concealed from the authorities and society. Families in which a member that has attempted suicide are afraid of being excommunicated and face severe social consequences [20]. People who suffer from mental illnesses are hesitant to seek psychiatric help due to the attached stigma [24]. Behaviors or disabilities that draw negative attention to the individual or the family are considered stigmatizing [25]. According to a report by the WHO, the Pakistani population has very poor access to psychiatric treatment facilities [26]. For a population exceeding 200 million people, there are only 343 registered psychiatrists, 478 psychologists, and 3,145 social workers in Pakistan [26]. Most of the Pakistani population live in rural areas, but most psychiatric services are available in urban settings.

Studies show that most suicides in Pakistan are committed by young people under age 30, with a greater incidence in women than men. The rates of suicide are highest among married women, surpassing the rates in both single women and married men. This contradicts results from the Western countries where marriage has been shown as a protective factor against suicide. For women in Pakistan, marital conflict and family structure is a clear stressor and a considerable source of psychological pressure [1, 20].

A matched case-controlled psychological autopsy study reported overwhelming evidence of an association between depression and suicide after adjusting for education, employment, and marital status; depression was the principal diagnosis on autopsy [21]. The rates of depression are high in population-based studies from Pakistan due to the higher prevalence of socioeconomic challenges. Depression and other mental health problems are often undiagnosed and untreated in Pakistan, which likely contributes to the suicide rates in the country [27].

The unstable political backdrop, worsening economic conditions, and a general lack of public faith in the governments and institutions have given rise to a sense of apprehension and misguidance regarding mental health. These problems render the traditionally protective aspects of culture and religion futile and can increase the rates of distress and depression which lead to suicides [27]. However, even with the recent awareness that poverty and stressful economic conditions impact the suicide rate, an emphasis on traumatic interpersonal relationships and psychiatric illnesses, especially depression, is crucial [27].

## Conclusions

To reduce the burden of suicide and decrease the associated stigma, there must be a strong, nationwide call for the decriminalization of suicide. While Islam condemns those who commit suicide, there are no clearly indicated legal and societal punishments in the Quran for those who survive suicide attempts. Religions can govern morality and dictate a code of conduct, but religious principles must be used carefully if used in the formulation of criminal laws. Using religion as a basis for devising legislations is a delicate issue and needs careful consideration. Research shows an underinvestigated, yet clear, underlying neuropathophysiology in those who commit suicide. If suicide is proven to be a byproduct of neurobiological and genetic interplay, it may not be any different from other mental health disorders triggered by stressful stimuli. To penalize patients who might be victims of a pathophysiology and to declare them as criminals is contrary to the compassionate nature of health care. Therefore, while shaping the laws related to suicide, it is imperative that legal stakeholders understand the role of mental health in suicidal behavior.  

The lack of a liaison between mental health care providers and the legal systems, a fear of persecution by the police, and primary health care workers' inability to recognize suicidal signs and conduct risk assessments for high-risk patients all contribute to a deteriorating suicide crisis. Unless the stigma surrounding suicide is removed by decriminalizing it, it is unlikely that Pakistan will be able to recognize, regulate, or assist those in need of professional help.
